# Aortic dissection after transcatheter aortic valve replacement

**DOI:** 10.1002/ccr3.2353

**Published:** 2019-08-04

**Authors:** Beatriz Martinez Quintero, Matthew R. Voss

**Affiliations:** ^1^ Department of Internal Medicine Saint Agnes Healthcare Baltimore MD USA

**Keywords:** cardiothoracic surgery, cardiovascular disorders, critical care medicine, emergency medicine

## Abstract

Aortic dissection is a rare but life‐ threatening complication of transcatheter aortic valve replacement, clinicians should be aware of this complication and should consider timely diagnostic evaluations, as well as, establish a prompt treatment plan based on a multidisciplinary team approach.

A 94‐ year‐ old man with NYHA class III heart failure who underwent transcatheter aortic valve replacement (TAVR) with a 34  mm Evolut R self‐ expandable heart valve (Medtronic, Inc) for severe aortic stenosis (AS) 3 _months prior presentation, presented with dyspnea and confusion. An unenhanced computed tomography of the chest showed the aortic prosthetic valve in place along a prominent aneurysmal dilation of the ascending thoracic aorta and internal displacement of mural calcifications toward the aortic lumen, suggestive of a Stanford type A aortic dissection (Figure 1, panel A, B). A transthoracic echocardiogram showed a severely dilated aorta with a complex intimal flap consistent with an aortic dissection (Figure 1, panel C, D). Due to his advanced age and high surgical risk, his family decided to forgo any surgical intervention and transition care toward comfort care.

**Figure 1:**
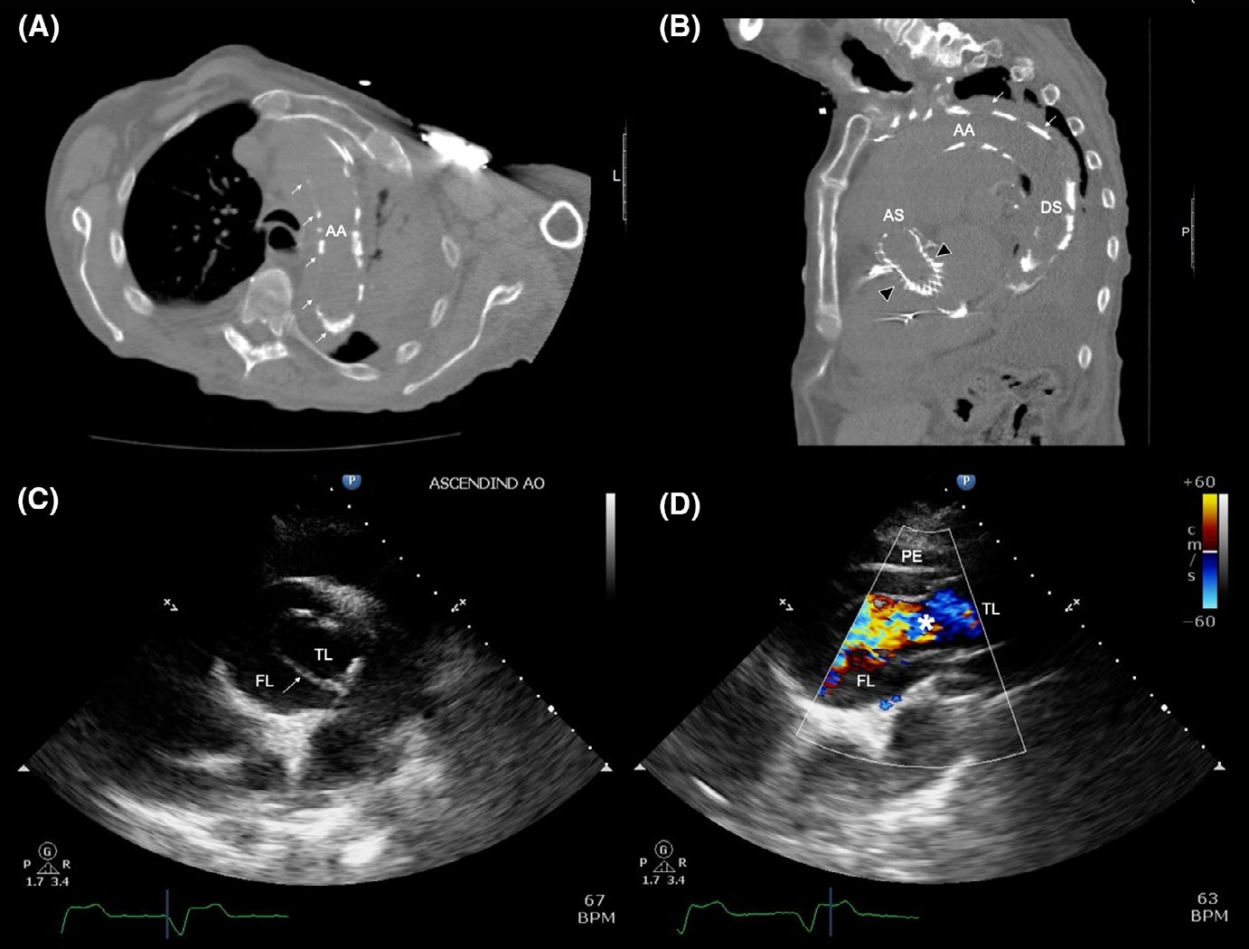
(A) Axial and, (B) Sagittal view of an unenhanced computed tomography (CT) of the chest showing a prominent aneurysmal dilation of the thoracic aorta with severe atherosclerotic vascular calcifications and internal displacement of mural calcifications toward the aortic lumen (arrows), suggestive of a Stanford type A aortic dissection; and demonstration of a prosthetic aortic valve in appropriate location (black arrowheads). AA, aortic arch; AS, ascending aorta; DS, descending aorta. Transthoracic echocardiogram, (C) parasternal short‐ axis view, and (D) parasternal long‐ axis view showing a severely dilated ascending aorta with a complex intimal flap (arrow) that divides the aorta into two, true and false, lumen; and (D) demonstrates the true lumen by color Doppler (asterisks) and a pericardial effusion. FL, false lumen; PE, pericardial effusion; TL, true lumen

Transcatheter aortic valve replacement has replaced surgical aortic valve replacement as the treatment of choice for symptomatic severe AS in patients at high surgical risk. Aortic dissection (AD) is a life‐ threatening complication of TAVR, reported in 0.6%‐ 1.9% of cases.^1^ Conventionally, the treatment of AD is emergent open surgical repair; however, its management in TAVR patients is challenging because they are already at high operative risk. More recently, less invasive procedures have gained popularity. Yet, the lack of evidence‐ based data and guidelines on its management makes it a treatment dilemma.

## CONFLICT OF INTEREST

None declared.

## AUTHOR CONTRIBUTIONS

All authors have made a substantial contribution to the preparation of this manuscript. BMQ: wrote the initial draft. MRV: provided expertise in image interpretation. All authors participated in the acquisition of the images for this manuscript, analysis and interpretation of the data, and critical revision of the manuscript for important intellectual content. All authors reviewed the final version of the manuscript and approved its submission.
